# Reduced toxicity matched sibling bone marrow transplant results in excellent outcomes for severe congenital neutropenia

**DOI:** 10.3389/fimmu.2024.1369243

**Published:** 2024-02-26

**Authors:** Joseph H. Oved, Nora M. Gibson, Kimberly Venella, Caitlin W. Elgarten, Lisa Wray, Julia T. Warren, Timothy S. Olson

**Affiliations:** ^1^ Division of Pediatric Transplantation & Cellular Therapies, Memorial Sloan Kettering Cancer Center, New York, NY, United States; ^2^ Division of Oncology, Department of Pediatrics, Children’s Hospital of Philadelphia, Philadelphia, PA, United States; ^3^ Division of Hematology, Department of Pediatrics, Children’s Hospital of Philadelphia, Philadelphia, PA, United States

**Keywords:** severe congenital neutropenia, stem cell transplant, conditioning regimen, busulfan and fludarabine, bone marrow failure, primary immunodeficiency

## Abstract

Severe congenital neutropenia (SCN) is caused by germline mutations, most commonly in *ELANE*, impacting neutrophil maturation and leading to high risk of life-threatening infections. Most patients with *ELANE-*mutant SCN can achieve safe neutrophil counts with chronic Granulocyte-Colony Stimulating Factor (G-CSF). However, up to 10% of patients have neutropenia refractory to G-CSF and require allogeneic stem cell transplant. Traditional conditioning for these patients includes busulfan and cyclophosphamide which is associated with significant toxicities. We present five patients with SCN without myeloid malignancy transplanted using a reduced toxicity regimen of busulfan, fludarabine and thymoglobulin. 5 pediatric patients with SCN underwent matched sibling donor bone marrow transplant (MSD-BMT) between 2014-2022 on or per CHP14BT057 (NCT02928991), a prospective, single center trial testing elimination of cyclophosphamide from conditioning in pediatric patients with single lineage inherited BMF syndromes. All patients had MSDs and no evidence of MDS. Conditioning consisted of PK-adjusted busulfan, fludarabine, and thymoglobulin, with calcineurin inhibitor and mycophenolate mofetil GVHD prophylaxis. With median follow-up of 48.4 months, overall and event-free survival were 100%. There was no acute GVHD and one instance of chronic limited GVHD. Patients exhibited >95% donor myeloid chimerism at 5 years post-BMT. Two patients experienced CMV reactivation without end-organ disease, and no other viral reactivation or significant infections occurred. MSD-BMT with reduced toxicity myeloablation for SCN provides excellent outcomes while minimizing toxicity. These data suggest that busulfan, fludarabine, and ATG can be considered an efficacious, low-toxicity standard of care regimen for patients with SCN undergoing MSD-BMT.

## 1 Introduction

Severe congenital neutropenia (SCN) defines a rare, heterogeneous group of disorders of neutrophil maturation. Prevalence estimates range from 1-9 cases per million individuals (1). More than thirty genes have been identified in which variants impair neutrophil differentiation, though this is not a universal finding (2–4). The most common of these are heterozygous *ELANE* mutations, accounting for approximately 50-60% of cases of SCN (5, 6). *ELANE* encodes neutrophil elastase, a serine protease enzyme contained in neutrophil azurophilic granules that hydrolyzes cell surface proteins and is released during the neutrophil activation process (6–8). Approximately two hundred distinct *ELANE* mutations have been identified, and the correlation of specific mutations to SCN phenotype are beginning to be understood (9, 10).

SCN typically presents with multiple severe infections in infancy and early childhood. These include omphalitis, pneumonia, bacteremia, abscess, and otitis media. Patients also frequently experience aphthous stomatitis and periodontitis. Historically, SCN had an approximate mortality rate of 50% in the first year of life due to sepsis, with a subsequent mortality of 6-7% per year. Since the advent of G-CSF therapy, patients with SCN have experienced a significant improvement in average lifespan and the mortality rate has been reduced to 0.9% per year (11). Patients with ELANE-mediated SCN on long-term G-CSF, however, have a cumulative risk of 22% for progression to MDS/leukemia after 15 years of treatment (11). Despite this leukemogenic risk, G-CSF is the initial treatment of choice, with a goal of achieving ANCs exceeding 1.0x10^9^/L (12). Approximately 10% of patients are unable to maintain this ANC and remain at risk for morbidity and mortality due to sepsis (13). Allogeneic stem cell transplant (AlloSCT) is the only curative treatment option for SCN. Definitive reasons to pursue HSCT include failure to maintain appropriate ANC with G-CSF or progression to MDS/leukemia. The cumulative risk of malignancy with G-CSF raises questions regarding the advisability of pre-emptive transplant for patients with high G-CSF requirements, recurrent intractable infections, or at some centers high-risk *ELANE* variants (9). In cases where a MSD is available, transplant is often considered. The appropriateness of pre-emptive alloSCT for patients with only alternative donor options is less clear (14, 15).

Given the rarity of SCN, there remains a paucity of data on the most appropriate conditioning regimen. To date, most patients who have had alloSCT for SCN have received myeloablative conditioning with busulfan and cyclophosphamide (Bu/Cy), and a small minority have received varied other regimens (16). This Bu/Cy myeloablative regimen carries numerous well-documented risks, including increased risk of veno-occlusive disease, pulmonary toxicity, infertility, and secondary malignancy (17). Busulfan and fludarabine has been proposed in this patient population as an alternative conditioning that can mitigate the severe side effects associated with cyclophosphamide (18–21). Our institutional experience supports this regimen, and we currently have an open phase I clinical trial to assess the efficacy of a busulfan/fludarabine-based conditioning regimen in bone marrow failure (BMF) patients. We present five pediatric patients with SCN transplanted using a conditioning regimen of busulfan, fludarabine, and ATG.

## 2 Methods

### 2.1 Patient characteristics

Five patients with *ELANE*-mediated SCN received MSD-BMT between 2014 and 2022 at our institution. Patient characteristics are listed in **Table 1**. All patients met phenotypic criteria for *ELANE*-related SCN prior to transplant, including ANC <0.2x10^9^/L in the absence of G-CSF treatment and a pathogenic *ELANE* variant. All patients were treated on or per CHP14BT057 (NCT02928991), a prospective, single center trial testing elimination of cyclophosphamide from conditioning in pediatric patients with single lineage BMF syndromes.

**Table 1:** Patient characteristics.

Patient	Age at Diagnosis (years)	*ELANE* Mutation	Age at Transplant (years)	Time from Diagnosis to Transplant (years)	ANC Baseline (prior to GCSF)	Prior Chronic GCSF (Y/N)	Pre-Transplant GCSF Dose (mcg/kg/day)	Pre-Transplant Infection History	Transplant Indication
1	0.4	Heterozygous c.607G>C	1.4	1.1	192	Y	12	Mastoiditis with TM perforation and hearing loss	High GCSF need
2	1.4	Heterozygous c.617C>T	2.4	1	0	Y	15	Skin abscesses, otitis media with TM perforation	High GCSF need
3	0	Heterozygousc.640G>A	0.3	0.2	0	Y	66	Omphalitis	GCSF refractory
4	3.9	Heterozygousc.242G>C	18.9	15	180	Y	5	Recurrent pulmonary infections Recurrent otitis media Cervical lymphadenitis	Pt preference
5	0	Heterozygous c.629 G>T	1.2	1.2	0	Y	22.5	Skin abscesses	High GCSF need

### 2.2 Transplant characteristics

All patients received a bone marrow graft from a 10/10 MSD. G-CSF was discontinued prior to start of conditioning. All patients received busulfan, fludarabine and ATG. Busulfan was PK-adjusted, and starting doses are listed in **Table 2**. For patients <1 year at time of transplant and for those treated before 2016, busulfan was administered every 6 hours, with target AUC of 900-1500 μmol*min/L (3.69-6.16 mg*h/L). The dose was adjusted based on the first dose PK per institutional SOP. Based on change in institutional SOP, for patients >1 year of age treated in or after 2016, daily dosing of busulfan was used targeting an AUC of 3600-6000 μmol*min/L (14.8-24.6 mg*h/L). Fludarabine was given at a dose of 150 mg/m2 or 5.2 mg/kg for infants <10kg over 4 days for patients treated before 2016, and 150 mg/m2 or 5 mg/kg for infants <10kg over 5 days for patients treated after 2016. This difference was due to change in institutional SOP. ATG dose was either 4.5 or 9 mg/kg, with difference again based on era of transplant due to a change in institutional SOP. The full conditioning schedule is listed in **Table 2**. For GVHD prophylaxis, all patients received IV cyclosporine (CsA) infusion followed by transition to oral tacrolimus once engraftment was achieved for a minimum of 3 months followed by taper. Patients also received mycophenolate (MMF) for a duration up to 45 days. Time to neutrophil and platelet engraftment was assessed per CIBMTR criteria. Peripheral blood donor chimerism was assessed by variable nucleotide tandem repeat analysis methods per institutional standard practice.

**Table 2:** Conditioning regimen.

Patient	ATG Total (mg/kg)	ATG Timing (Day)	Fludarabine Total	Fludarabine Timing (Day)	Busulfan Start/Cumulative Dose (mg/kg)	Busulfan Dose Sched	Busulfan Cumulative Dose AUC* (μmol*min/L)	Busulfan Timing (Day)	GVH/Rejection Prophylaxis
1	4.5	-10 to -8	5.2 mg/kg	-5 to -2	0.8/16.3	q6h	1197	-7 to -4	CsA/Tacro/MMF
2	4.5	-10 to -8	150 mg/m2	-5 to -2	1/16.1	q6h	906	-7 to -4	CsA/Tacro/MMF
3	9	-10 to -8	5 mg/kg	-6 to -2	0.8/12.9	q6h	1050	-7 to -4	CsA/Tacro/MMF
4	9	-10 to -8	150 mg/m2	-6 to -2	3.2/14.7	daily	4573	-7 to -4	CsA/Tacro/MMF
5	9	-10 to -8	5 mg/kg	-6 to -2	3.2/18.4	daily	4237	-7 to -4	CsA/Tacro/MMF

## 3 Results

### 3.1 Disease history

*ELANE* variants for the 5 patients with SCN undergoing MSD-BMT are listed in **Table 1**, along with age at diagnosis, baseline ANC ranges (prior to G-CSF initiation) and history of pre-transplant complications. Notable pre-transplant infections included: omphalitis, recurrent otitis media, mastoiditis, skin infections, pulmonary infections, and lymphadenitis. No patients had evidence of MDS or leukemia at the time of transplant. Weight based G-CSF dosing at the time of transplant ranged from 5 mcg/kg/day to 66 mcg/kg/day. The indications for transplant included need for high doses of G-CSF (n=3), failure to achieve safe ANC with G-CSF therapy (n=1) and patient preference (n=1). Age at time of BMT ranged from 0.3-18.9 years.

### 3.2 Engraftment, survival, and donor chimerism

**Figure 1:**
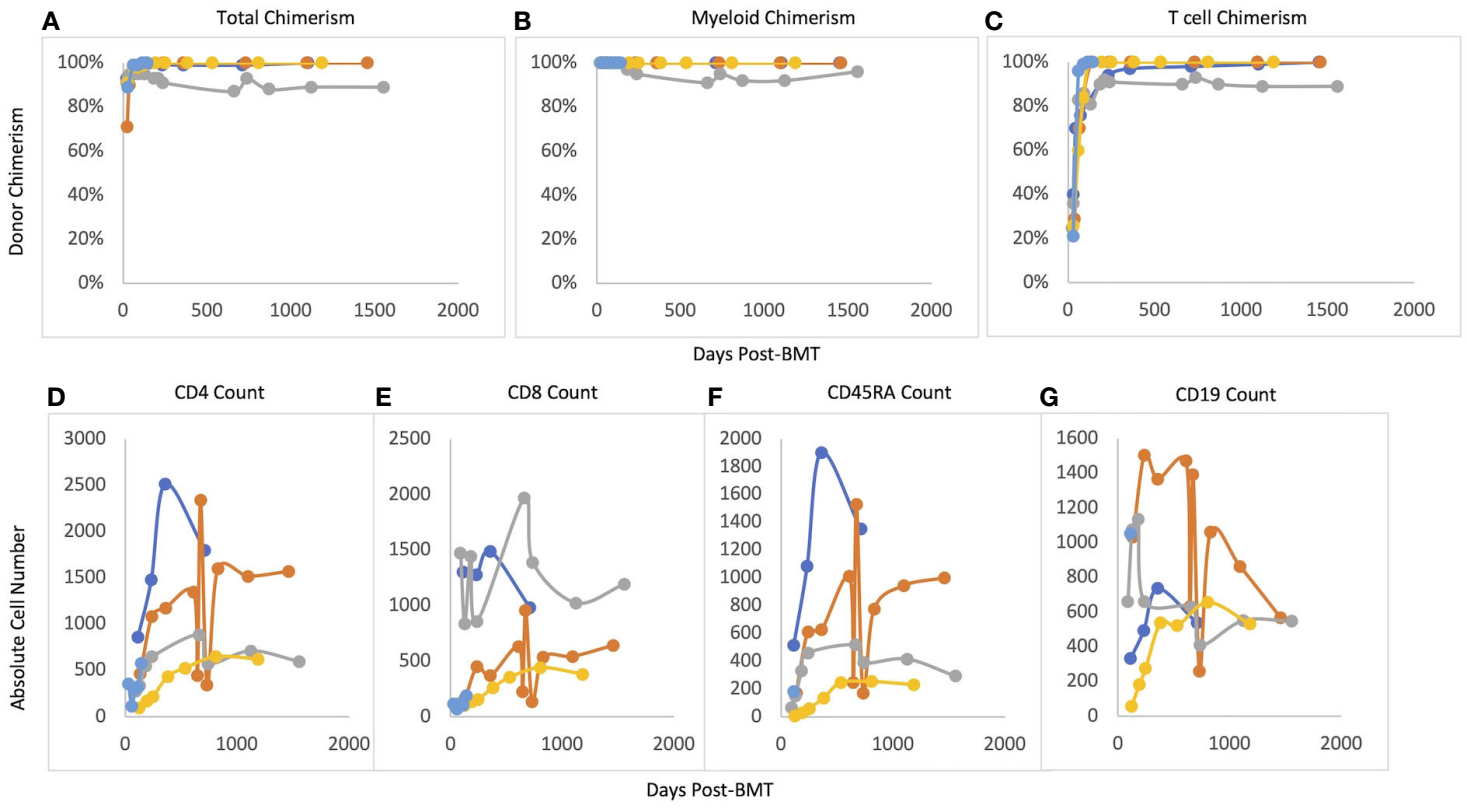
Post-BMT chimerism and immune reconstitution. **(A)** Total chimerism, **(B)** Myeloid chimerism, **(C)** T cell chimerism, **(D)** CD4 reconsitution; **(E)** CD8 reconstitution, **(F)** CD45RA reconstitution, **(G)** CD19 reconstitution.

Median post-transplant follow-up ranged was 48.4 months (12-51.9 months). Median time to neutrophil engraftment was 21 days (16-38 days) (see **Table 3**). Two patients received G-CSF starting at Day +15 and Day +17 based on clinician preference and was continued for 4 days before stopping based on engraftment. Median time to platelet engraftment was 19 days (16-30 days). Overall and event-free survival were 100%. No patient required G-CSF after engraftment. All patients reached at least 95% total donor chimerism by 60 days after transplant and maintained >90% total chimerism throughout their entire period of follow-up (**Figure 1**). All patients reached 100% donor myeloid chimerism by day 36 after transplant and maintained >95% chimerism for the remainder of follow-up. T cell chimerism increased consistently in the first six months after transplant and remained at or above 89% in all patients.

**Table 3:** Results and transplant complications.

Patient	Follow-up Time (months)	Time to Neutrophil Engraftment (days)	Time to Platelet Engraftment (days)	Total Chimerism at ~60 days After Transplant (%)	Acute GVHD (Y/N)	Chronic GVHD (Y/N)	Viral Reactivation (Y/N)	Bacterial and Fungal Infections
1	47.7	16	16	95	N	N	Y, CMV	None
2	48.0	21	17	97	N	Y (limited)	N	None
3	51.2	21	28	96	N	N	Y, CMV	*E. faecalis* bacteremia
4	39.0	38	30	98	N	N	N	None
5	4.7	17	19	99	N	N	N	None

### 3.3 GVHD, infections, and other adverse events

There were no cases of acute GVHD. One patient developed chronic limited GVHD which manifested as fasciitis limited to hands and wrists. These symptoms were treated with a course of prednisone and imatinib with full symptom resolution. No other patients required systemic GVHD therapy. Two patients developed low-level CMV reactivation managed with anti-viral medications. Neither developed CMV disease and time to resolution of CMV was 53 days post-transplant. There were no other viral reactivations. Bacterial and fungal infections during the peri- and post-transplant periods included *E. faecalis* bacteremia in one patient. No severe organ complications occurred. One patient had transient electrolyte wasting without evidence of renal dysfunction. No patients developed hepatic veno-occlusive disease, transplant-associated thrombotic microangiopathy, or chronic lung function impairment.

## 4 Discussion

*ELANE*-related SCN is a rare disorder of neutrophil maturation that carries a high risk of life-threatening infections. It requires lifelong G-CSF therapy to mitigate this risk, but G-CSF may cause severe long-term side effects, including significant osteopenia, splenomegaly, and risk of myeloid malignancies. AlloSCT is the only currently available curative therapy for SCN. The discussion to consider alloSCT becomes more acute in patients who develop clonal evolution involving somatic *RUNX1* mutations or cytogenetic abnormalities and many centers will consider using best available alternative donor in these cases. Thus, the question of which patients should consider pre-emptive transplant in the absence of myeloid malignancies is much debated. Most centers consider patients who require high standing doses of G-CSF (≥8 mcg/kg/day) and who have a suitable available MSD to be excellent candidates for pre-emptive transplant, but whether this approach should be offered to patients with lower daily G-CSF requirements or for those whose options are limited to alternative donor transplant is less certain.

There are no defined standards of care for conditioning in alloSCT for *ELANE*-related SCN. Busulfan and cyclophosphamide is the most common regimen used in published cases (16), but poses significant risks from dual alkylating agents. We present 5 patients that received MSD-BMT using a reduced toxicity busulfan and fludarabine conditioning regimen for *ELANE*-mutant SCN without myeloid malignancy. None of the patients in this study developed significant lung, liver, or kidney toxicity, and overall survival was 100%. This strategy also did not compromise efficacy. All patients exhibited event-free survival, time to neutrophil and platelet engraftment and total donor chimerism comparable to busulfan and cyclophosphamide-based regimens (21–23). Our patients exhibited low rates of GVHD with no cases requiring ongoing systemic therapy. Viral reactivation occurred in a minority of patients and resolved without end-organ damage in all cases.

MSD-BMT with myeloablative but reduced-toxicity conditioning for *ELANE*-mutant SCN provides excellent outcomes with minimal toxicity. Additional reduced-toxicity regimens could also be considered, including treosulfan/fludarabine. Treosulfan has demonstrated comparable efficacy and a favorable toxicity profile compared to busulfan (24–26), and may represent an opportunity to further optimize outcomes. However, the currently enrolling clinical trial BMT-CTN 1904 examining a treosulfan-based conditioning regimen for patients with bone marrow failure excludes patients with SCN due to a paucity of data using similar regimens including busulfan/fludarabine. As our study demonstrates excellent engraftment for a busulfan/fludarabine/ATG regimen, we theorize that treosulfan might perform similarly and should be tested in patients with SCN.

Our study is limited in scope by the small size and lack of control group receiving traditional busulfan/cyclophosphamide regimens for comparison; however, comparisons to cases in the literature are favorable. These pilot data suggest that this busulfan and fludarabine-based approach should be studied in multicenter clinical trials, where any patient with *ELANE*-related SCN who has a MSD and daily G-CSF requirement would be eligible.

## Data availability statement

The original contributions presented in the study are included in the article/supplementary material. Further inquiries can be directed to the corresponding author.

## Ethics statement

The studies involving humans were approved by Children’s Hospital of Philadelphia Institutional Review Board. The studies were conducted in accordance with the local legislation and institutional requirements. Written informed consent for participation in this study was provided by the participants’ legal guardians/next of kin.

## Author contributions

JO: Writing – original draft, Writing – review & editing, Conceptualization, Methodology. NG: Writing – original draft, Writing – review & editing, Data curation, Formal analysis. KV: Investigation, Writing – review & editing, Resources, Project administration. CE: Conceptualization, Resources, Supervision, Writing – review & editing. LW: Conceptualization, Resources, Writing – review & editing. JW: Supervision, Writing – review & editing. TO: Conceptualization, Supervision, Writing – original draft, Writing – review & editing, Methodology.

## References

[1] Donadieu J Beaupain B Mahlaoui N Bellanné-Chantelot C. Epidemiology of congenital neutropenia. Hematol Oncol Clin North Am. (2013) 27:1–17. doi: 10.1016/j.hoc.2012.11.003 23351985

[2] Donadieu J Bellanné-Chantelot C. Genetics of severe congenital neutropenia as a gateway to personalized therapy. Hematol Am Soc Hematol Educ Program. (2022) 2022:658–65. doi: 10.1182/hematology.2022000392 PMC9821599 36485107

[3] Willemsen M Barber JS Nieuwenhove EV Staels F Gerbaux M Neumann J. Homozygous DBF4 mutation as a cause of severe congenital neutropenia. J Allergy Clin Immunol. (2023) 152:266–77. doi: 10.1016/j.jaci.2023.02.016 36841265

[4] Linder MI Mizoguchi Y Hesse S Csaba G Tatematsu M Lyskiewicz M. Human genetic defects in SRP19 and SRPRA cause severe congenital neutropenia with distinctive proteome changes. Blood. (2023) 141:645–58. doi: 10.1182/blood.2022016783 PMC10651786 36223592

[5] Rosenberg PS Alter BP Link DC Stein S Rodger E Bolyard AA. Neutrophil elastase mutations and risk of leukaemia in severe congenital neutropenia. Br J Haematol. (2008) 140:210–3. doi: 10.1111/j.1365-2141.2007.06897.x PMC3143022 18028488

[6] Horwitz MS Duan Z Korkmaz B Lee HH Mealiffe ME Salipante SJ. Neutrophil elastase in cyclic and severe congenital neutropenia. Blood. (2007) 109:1817–24. doi: 10.1182/blood-2006-08-019166 PMC1801070 17053055

[7] Horwitz M Benson KF Person RE Aprikyan AG Dale DC. Mutations in ELA2, encoding neutrophil elastase, define a 21-day biological clock in cyclic haematopoiesis. Nat Genet. (1999) 23:433–6. doi: 10.1038/70544 10581030

[8] Skokowa J Dale DC Touw IP Zeidler C Welte K. Severe congenital neutropenias. Nat Rev Dis Primers. (2017) 3:17032. doi: 10.1038/nrdp.2017.32 28593997 PMC5821468

[9] Makaryan V Zeidler C Bolyard AA Bonilla MA Boxer LA Cham B. The diversity of mutations and clinical outcomes for ELANE-associated neutropenia. Curr Opin Hematol. (2015) 22:3–11. doi: 10.1097/moh.0000000000000105 25427142 PMC4380169

[10] Germeshausen M Deerberg S Peter Y Reimer C Kratz CP Ballmaier M. The spectrum of ELANE mutations and their implications in severe congenital and cyclic neutropenia. Hum Mutat. (2013) 34:905–14. doi: 10.1002/humu.22308 23463630

[11] Rosenberg PS Alter BP Bolyard AA Bonilla MA Boxer LA Cham B. The incidence of leukemia and mortality from sepsis in patients with severe congenital neutropenia receiving long-term G-CSF therapy. Blood. (2006) 107:4628–35. doi: 10.1182/blood-2005-11-4370 PMC1895804 16497969

[12] Bonilla MA Gillio AP Ruggeiro M ernan NA Brochstein JA Abboud M. Effects of recombinant human granulocyte colony-stimulating factor on neutropenia in patients with congenital agranulocytosis. N Engl J Med. (1989) 320:1574–80. doi: 10.1056/nejm198906153202402 2471075

[13] Dale DC Bolyard AA Shannon JA Connelly JA Link DC Bonilla MA. The severe chronic neutropenia international registry: 10-year follow-up report. Support Cancer Ther. (2006) 3:220–31. doi: 10.3816/SCT.2006.n.020 18632498

[14] Choi SW Levine J. Indications for hematopoietic cell transplantation for children with severe congenital neutropenia. Pediatr Transplant. (2010) 14:937–9. doi: 10.1111/j.1399-3046.2010.01386.x 20819181

[15] Connelly JA Walkovich K. Diagnosis and therapeutic decision-making for the neutropenic patient. Hematol Am Soc Hematol Educ Program. (2021) 2021:492–503. doi: 10.1182/hematology.2021000284 PMC8791128 34889413

[16] Connelly JA Choi SW Levine JE. Hematopoietic stem cell transplantation for severe congenital neutropenia. Curr Opin Hematol. (2012) 19:44–51. doi: 10.1097/MOH.0b013e32834da96e 22080845 PMC3291495

[17] Ben-Barouch S Cohen O Vidal L Avivi I Ram R. Busulfan fludarabine vs busulfan cyclophosphamide as a preparative regimen before allogeneic hematopoietic cell transplantation: systematic review and meta-analysis. Bone Marrow Transplant. (2016) 51:232–40. doi: 10.1038/bmt.2015.238 26457908

[18] Hashem H Abu-Arja R Auletta JJ Rangarajan HG Varga E Rose MJ. Successful second hematopoietic cell transplantation in severe congenital neutropenia. Pediatr Transplant. (2018) 22. doi: 10.1111/petr.13078 29076228

[19] Carlsson G Winiarski J Ljungman P Ringden O Mattsson J Nordenskjold M. Hematopoietic stem cell transplantation in severe congenital neutropenia. Pediatr Blood Cancer. (2011) 56:444–51. doi: 10.1002/pbc.22836 21072829

[20] Oshima K Hanada R Kobayashi R Kato K Nagatoshi Y Tabuchi K. Hematopoietic stem cell transplantation in patients with severe congenital neutropenia: an analysis of 18 Japanese cases. Pediatr Transplant. (2010) 14:657–63. doi: 10.1111/j.1399-3046.2010.01319.x 20331518

[21] Rotulo GA Beaupain B Rialland F Paillard C Nachit O Galambrun C. HSCT may lower leukemia risk in ELANE neutropenia: a before-after study from the French Severe Congenital Neutropenia Registry. Bone Marrow Transplant. (2020) 55:1614–22. doi: 10.1038/s41409-020-0800-1 PMC7091645 31992846

[22] Ferry C Ouachée M Leblanc T Michel G Notz-Carrere A Tabrizi R. Hematopoietic stem cell transplantation in severe congenital neutropenia: experience of the French SCN register. Bone Marrow Transplant. (2005) 35:45–50. doi: 10.1038/sj.bmt.1704718 15489867

[23] Zeidler C Welte K Barak Y Barriga F Bolyard AA Boxer L. Stem cell transplantation in patients with severe congenital neutropenia without evidence of leukemic transformation. Blood. (2000) 95:1195–8. doi: 10.1182/blood.V95.4.1195.004k36_1195_1198 10666190

[24] van der Stoep M Bertaina A Moes D Algeri M Bredius RGM Smiers FJW. Impact of treosulfan exposure on early and long-term clinical outcomes in pediatric allogeneic hematopoietic stem cell transplantation recipients: A prospective multicenter study. Transplant Cell Ther. (2022) 28:99.e1–7. doi: 10.1016/j.jtct.2021.09.018 34607071

[25] Olivas-Mazón R Bueno D Sisinni L Mozo Y Casado-Abad G Martínez AP. A retrospective study of treosulfan versus busulfan-based conditioning in pediatric patients. Eur J Haematol. (2022) 109:474–82. doi: 10.1111/ejh.13828 35810360

[26] Suh JK Im HJ Kang SH Kim H Choi ES Koh KN. Treosulfan-based conditioning regimen for allogeneic hematopoietic stem cell transplantation in children with non-malignant diseases. Bone Marrow Transplant. (2022) 57:681–4. doi: 10.1038/s41409-021-01556-8 35132202

